# THE APPLICATION OF USER-CENTERED DESIGN TO A DIGITAL PLATFORM FOR OLDER ADULTS: THE PRISM SOFTWARE SYSTEM

**DOI:** 10.1093/geroni/igae098.1842

**Published:** 2024-12-31

**Authors:** Sara Czaja, Walter Boot, Wendy Rogers, Neil Charness

**Affiliations:** Weill Cornell Medicine/Center on Aging and Behavioral Research, New York, New York, United States; Weill Cornell Medicine, New York, New York, United States; University of Illinois Urbana-Champaign, Champaign, Illinois, United States; Florida State University, Tallahassee, Florida, United States

## Abstract

Technology holds promise for enhancing the social connectivity and reducing loneliness among older adults, especially those that are socially isolated. However, technology systems can be overly complex and challenging especially for those with limited technology experience. Common barriers to technology uptake among aging adults include usability issues, and lack of training and support. User-centered design is critical to maximize the uptake of supportive technologies among older adult users. This presentation will provide an example of how the user-centered design process was applied to the design of a digital platform for older adults, the PRISM software system. The iterative design approach and the incorporation of user feedback into the design process will be highlighted. PRISM, an integrated software system, was designed to foster social connectivity, cognitive engagement, and resource access among aging adults. Versions of PRISM have been evaluated in diverse contexts and geographic regions, and with diverse populations, including individuals with a cognitive impairment. Data will be presented regarding participants use of and perceptions of the usability and value of PRISM. Findings regarding the impact of the use of PRISM on social connectivity and perceptions of loneliness will also be presented. A focus will be on the adaptation of PRISM for aging adults with a cognitive impairment and strategies used to provide training for this population. Recommendations for needed future research in the aging and technology domain will also be provided to ensure that the emerging technology applications are designed to meet the needs, abilities, and preferences of aging adults.

